# Interviews with Patients and Providers on Transgender and Gender Nonconforming Health Data Collection in the Electronic Health Record

**DOI:** 10.1089/trgh.2016.0041

**Published:** 2017-02-01

**Authors:** M.J. Dunne, Lewis A. Raynor, Erika K. Cottrell, William J.A. Pinnock

**Affiliations:** ^1^ OCHIN, Inc., Portland, Oregon.; ^2^ Oregon Health & Science University, Portland, Oregon.

**Keywords:** data collection, electronic health records, health disparities, non-conforming, safety-net, transgender

## Abstract

**Purpose:** Meaningful use (MU) and Uniform Data Systems (UDSs) are calling for the collection of gender identity (GI) in electronic health record (EHR) systems; however, many transgender and nonconforming (TGNC) patients may not feel safe disclosing their GI and the data collection is not designed to guide care provision. This study explores the complexities surrounding the inclusion of GI in EHR data collection and how it can best serve patients and providers.

**Methods:** Using a semistructured interview format, TGNC patients (*n*=7) and providers (*n*=5) who care for TGNC patients were asked about data collection procedures and the use of these data within community health centers in Oregon. Using a constant comparative data analysis methodology, interview transcripts were coded for emergent concepts until overlapping themes were identified.

**Results:** Both patients and providers expressed a need for the EHR to expand upon MU and UDS-recommended fields to include current pronouns and name and gender identifiers in a forward-facing display to prevent misgendering by clinic staff and providers. Furthermore, they both cited the need for a broader range of birth-assigned sex and gender options. TGNC patients and providers disagreed on the scope of health information to be collected as well as who should be tasked with the data collection.

**Conclusion:** These interviews offer us a glimpse into the structural difficulties of creating an EHR system that serves the needs of clinicians while providing safe and culturally competent care to TGNC patients.

## Introduction

Numerous national organizations have noted the scarcity of and the need for transgender/gender nonconforming (TGNC) health research.^1–5^ Healthy People 2020 stated that a lack of gender identity (GI) data in national datasets has hindered efforts to accurately measure health needs and outcomes in TGNC populations and called for additional research on TGNC populations, within standardized data.^6^ What little research that has been done shows that this population faces disproportionate poverty, homelessness, and uninsurance/underinsurance.^7–9^ High levels of low socioeconomic status surely mean worse healthcare access, quality, and outcomes in TGNC populations; however, population-based epidemiology for this population is largely absent in the United States.

Beginning in 2016, the Uniform Data System (UDS) guidelines mandated the collection and reporting of GI for all health center program grantees and look-alikes.^10^ Meaningful use (MU) stage 3 has also called for all electronic health record (EHR) vendors to create systems that allow users to record, change, and access structured data on GI, but this change does not require providers to collect GI information.^11^ UDS and MU both call for GI data collection in the demographic fields; however, UDS and MU offer different methods of GI collection, different GI options, no guidelines are provided on who should collect these data, and neither program addresses how the collection of GI could and/or should affect clinical workflows and insurance billing.^10,12^

While national systems are calling for the collection of GI, TGNC patients may feel unsafe disclosing their GI because of healthcare discrimination.^2^ Research has shown that TGNC individuals face many barriers to receiving transition-related and nontransition-related healthcare, including a lack of access to culturally competent providers, fear of harassment by medical staff, and the outright refusal of care provision by some providers.^8^ With this important limitation to data collection in mind, we interviewed healthcare providers and TGNC patients to determine how and where GI should be captured in the EHR of community health centers (CHCs) in Oregon. The results of this exploratory research help create the development of best practices for collecting and documenting GI information in CHC settings.

## Background

### Transgender healthcare disparities and CHCs in Oregon

The 2011 National Transgender Discrimination Survey found that TGNC people are nearly four times more likely to live in very low-income households compared with the general population with unemployment twice that of the general population.^8^ The disproportionate impact of poverty and employment discrimination means that many TGNC people are uninsured or underinsured which may equate to worse healthcare access, quality, and outcomes in TGNC populations.^7,9^ The capture of GI within the EHR creates opportunities for TGNC health research, as evidenced by recent studies conducted by health systems such as the Veterans Health Administration and Kaiser Permanente that identified TGNC patients in the EHR and characterized their health outcomes.^13–16^ However, research using existing health systems may miss hard-to-reach TGNC population subgroups such as the uninsured.^17^ Because TGNC people are more likely to be low income and uninsured or insured through public programs, they are likely to access healthcare at CHCs serving economically disadvantaged populations, thus necessitating the development of systems to capture GI data in CHC settings.

### MU, UDS, and GI

The Institute of Medicine (IOM) 2011 report on the health of LGBT Americans noted that while healthcare disparities are well documented for the TGNC community, epidemiology for this population is largely absent in the United States. The dearth of research is partially rooted in a scarcity of GI data collection in healthcare systems, leading the IOM to call for the collection of GI data in the EHR as part of its MU requirements. The IOM noted that barriers to data collection include healthcare worker discomfort with collecting this information, healthcare workers not knowing how to ask for this information, and patient hesitancy to disclose GI status.^2^

In 2016, both the Centers for Medicare and Medicaid Services and the Office of the National Coordinator for Health Information Technology (ONC) announced that they will require all EHR systems certified under the MU incentive program to create fields that allow users to record, change, and access structured data on GI to be certified to the 2015 edition demographics certification criterion.^12,18^ ONC states that certification does not require a provider to collect this information; rather, it requires that the certified electronic health record technology enables the provider to do so.^18^ The Health Resources and Service Administration of the U.S. Department of Health and Human Services has also called for the collection of GI data in their UDS, which is a collection of performance measures required for health center program grantees and look-alikes.^10,12^

Both systems of data collection call for the capture of GI in the demographic fields, but diverge in how data are collected. ONC calls for the collection of both birth-assigned sex and GI and offers vocabulary standards. Birth-assigned sex is recorded as (a) male, (b) female, or (c) other. The minimal standard for GI includes the following options: (a) male; (b) female; (c) female-to-male/transgender male/transman; (d) male-to-female/transgender female/transwoman; (e) genderqueer, neither exclusively male nor female; (f) additional gender category or other, please specify; and (g) choose not to disclose.^18^

UDS captures birth-assigned sex in Table 1 (patients by age and sex) and GI in Table 2 (demographic characteristics). Gender options for patients include (a) male, (b) female, (c) transgender male/female-to-male, (d) transgender female/male-to-female, (e) other, and (f) choose not to disclose.^19^

**Table 1.** **Uniform Data System: Patients by Age and by Sex Assigned at Birth**

Line	Age groups	Male patients (a)	Female patients (b)
1	Under age 1		
2	Age 1		
…			
38	Ages 85 and over		
39	Total patients (sum lines 1–38)		

**Table 2.** **Uniform Data System: Patients by Gender Identity**

Line	Patients by gender identity	Number (a)
20	Male	
21	Female	
22	Transgender male/female-to-male	
23	Transgender female/male-to-female	
24	Other	
25	Choose not to disclose	
26	Total patients (sum lines 20 to 25)	

## Methods

### Study setting

OCHIN is a collaborative member-based organization of federally qualified health centers (FQHCs) and similar entities providing a primary healthcare safety net to vulnerable populations. Originally named the Oregon Community Health Information Network, this nonprofit, community-based, health information technology collaborative was renamed OCHIN when other states joined.^20,21^ Currently, OCHIN provides health information technology to >300 CHCs in 19 states that are a part of the OCHIN collaborative, and members share a single, fully integrated centrally hosted Epic^©^ EHR.^22^ In Oregon, OCHIN provides services to 22 of the 33 CHCs recognized by the Oregon Primary Care Association.^23^ OCHIN is committed to the CHC tradition of patient and clinician engagement, whereby their voices inform decisions made within the healthcare system.^24,25^

### Study population

We interviewed *n*=7 patients who self-identified as TGNC, were current residents of Oregon, were insured by Medicaid, and had received or were currently receiving care from an Oregon CHC. Patients were initially recruited by having a trusted member of the TGNC community post a request for research participation on a closed Facebook group serving the TGNC community. Subsequent patients were recruited using a snowball sampling methodology after gaining the trust of their peers. We interviewed *n*=5 providers who work in a CHC in Oregon and have at least one TGNC patient. Four of the clinics were located in Portland, Oregon, and one clinic was located in a rural town in southern Oregon. Providers were initially identified through the Oregon Health & Science University's Transgender Health Program, which was created to support access to welcoming and affirming healthcare for TGNC patients, and the Equi Institute, which is a Portland-based LGBTQI health clinic. Additional providers were recruited using convenience and snowball sampling after the initial interviewees were identified.

### Interviews

In-person, phone, and videoconferencing interviews were conducted to explore how current EHR data collection practices affect the health and well-being of TGNC patients and how these practices can be modified to meet the needs of both providers and patients. We utilized an exploratory research design to solicit patient and provider insights on EHR data collection practices for TGNC community members.^26^ This exploratory research is timely as FQHCs and look-alikes are starting the process of modifying their EHR systems to collect GI data as per UDS requirements. Both the provider and patient interviews followed a general script, but allowed the interviewer an opportunity to explore other topics (Table 3). Interview questions were developed with patient and provider feedback and further refined by researchers and members within a local LGBTQ community research group.

**Table 3.** **Provider and Transgender and Nonconforming Patient Interview Questions**

Provider/clinic interview questions	Transgender patient interview questions
Does your health center currently collect GI^a^ identity data in the EHR?	How long have you lived in Oregon?
– If not, do you currently have plans to collect this info?	– If you moved here recently, did the Medicaid coverage of TGNC-related care factor in your decision to move?
– If not, do you think your health center would benefit from the collection of GI data?	Where do you receive primary healthcare?
– If yes, who collects this information?	Does your clinic know your GI?
– If yes, did the individual who collects this information receive any training on GI and data collection?	– If not, why?
– If yes, where is it collected (progress note, structured field, demographics)?	– Do you think it is important for your clinic to know your GI?
– If yes, what kinds of GI data are collected (current name/pronouns, organ inventory, birth-assigned sex)?	Does your PCP know your GI?
What does your clinic do when the GI of the patient does not match the legal sex of the patient?	– If not, why?
Has the expansion of Medicaid coverage for gender transition-related care impacted your clinic?	– Do you think it is important for your PCP to know your GI?
– If yes, how?	Who do you feel most safe disclosing your GI to (front desk, medical assistant, physician)?
Do you have any TGNC patients?	How has disclosing your GI impacted your care at the CHC you currently see your PCP at?
Where do you think the collection of GI would be best utilized (demographics, structured field, searchable field) for your clinical care?	– Positives*?*
How has where GI is collected impacted your care provision?	– Negatives*?*
Has your patient's current GI caused problems with insurance billing?	How is your GI recorded by your insurance company?
If anatomy was not collected as part of the GI data collection, how did you go about ascertaining what screenings to enact for a patient?	Is your decision to disclose your GI impacted by how it is captured by your insurance provider?
Has the collection of GI changed your relationship with your patient/patient care provision?	How would you like your GI to be captured in a medical system?
– If so, how?	

^a^ The use of GI in this table captures gender and sex—terms which are often used interchangeably in social systems. For example, we asked TGNC patients about their current GI and insurance billing. Many insurance companies capture GI through sex fields.

CHC, community health center; EHR, electronic health record; GI, gender identity; PCP, primary care physician; TGNC, transgender and nonconforming.

The interviews lasted ∼15 to 60 min with a brief introduction to allow for verbal informed consent. The researchers took notes during and after the provider and patient interviews to ensure that all relevant details were accurately recorded. Patient interviews followed a script consisting of (1) an introduction to the researchers, (2) an introduction to the study, (3) questions to confirm inclusion criteria, (4) a series of questions to explore how the collection of personal health information (PHI) regarding an individual's GI has affected their healthcare, and (5) questions aimed at exploring best practices around the collection of GI information. Provider interviews followed the same semistructured format, with similar exploratory questions around the collection of PHI and GI, but with additional questions aimed at evaluating *how* GI is collected in their clinic's EHR system. It was important to interview providers with experience treating TGNC patients to better understand current clinical best practices in regard to TGNC health.

### Data analysis

Using a grounded theory methodology, semistructured patient interviews were coded for emergent concepts until overlapping themes were identified.^27,28^ Grounded theory methodology was used for this exploratory study as a way to give an active voice to a population that is hard to reach or marginalized.^28,29^ Grounded theory is useful in building theoretical concepts about patient engagement with healthcare systems when the topic of interest has not been thoroughly studied.^30^

A constant comparative data analysis method was used to analyze the interview data.^29^ The researchers extracted sections of the interview data from interview transcripts and notes and coded emergent thematic elements. Each subsequent interview was coded and then compared with the previously categorized data elements until no new themes emerged. This study was approved by the institutional review board and received an exemption determination as no PHI was collected during the interviews.

## Results

### What do providers and transgender patients want from the EHR to improve quality of care?

Both patients and providers would like the EHR system to have pronouns, name (even if different from legal name), and gender identifiers viewable by front-line staff to prevent misgendering and misnaming by healthcare providers and staff (Table 4). Several patients had experiences of being misgendered in a clinic waiting room or exam room. One patient remarked that he was often referred to as Miss in the waiting room despite his medical provider having the current gender recorded in other sections of the EHR. Patients stated the need for their current name, whether legally changed or not, to be captured in the EHR and viewable to all clinic staff. Deadnaming is the practice of using the name a TGNC individual used before transitioning, thereby invalidating a person's GI.^31^ Patients said that having their deadname called out in clinical settings was upsetting. One patient noted that while his name was listed in his chart, clinic staff repeatedly called him by his deadname. Another patient said, “Having a provider not call me by my deadname would be awesome.”

**Table 4.** **Key Research Findings**

What do providers and transgender patients want from an EHR system?	Where do providers and transgender patients differ in opinion?
• Both want preferred pronouns, preferred name, and gender identifier in a forward-facing display.	• Patients felt strongly that they should not have to divulge birth-assigned sex unless they wanted to. Physicians and clinicians felt this was crucial information to document for primary care.
• Patients expressed the need for a broader range of gender and birth-assigned sex identifiers (e.g., agender, nonbinary, and intersex).	• Patients and providers had differing opinions on who should be tasked with collecting TGNC health information.

Clinicians agreed that having current names in a forward-facing display would likely improve care and help alleviate unintentional misgendering by clinic staff and themselves. One physician commented that when the gender pronouns and the patient's name are appropriately captured in the EHR and used at each visit, it becomes less of an issue and normalizes the interaction. Providers also noted that this is complicated by existing EHR structures that do not necessarily allow clinics to capture GI appropriately, creating the need for workarounds. For example, a patient's current gender may not match their legal gender, which can cause billing systems to function inappropriately.

Finally, providers and patients agreed that the EHR needs to include a broader range of birth-assigned sex and gender options. Two patients stated that they are intersex, and they both wished there was an option to choose intersex as a birth-assigned sex category. One patient stated, “Being able to check ‘male’ or ‘female’ may make it easier when going to the DMV, but every time I check ‘male’ or ‘female’ on paperwork I feel like I'm lying.” This was especially true for those patients who identified as gender nonconforming. One patient said, “You will have people who don't want ‘trans’ in there at all. My roommate identifies as ‘agender.’” Another said, “I have passing privilege and can blend in, but there is a community of nonbinary people who may not identify as male or female and they are OK with that.” One patient who identified as genderqueer states, “It's important that if they have a section that says ‘M'am’ or ‘Sir,’ then they also have an option to choose ‘none of these.’”

### Where do providers and transgender patients differ in opinion in regard to EHR data collection?

Patients and providers disagreed on the scope of health information that should be collected as well as who should be tasked with the data collection. TGNC patients felt strongly that they should not have to divulge their birth-assigned sex unless it was absolutely necessary for care provision. One patient said, “If seeing a new doctor, I don't need that to be the first thing they know about me. I should be able to disclose at my discretion. From my experience that is all I become—nothing more than their ‘transgender patient.’ I am more than just my trans identity.” Another patient said, “I know from personal experience that doctors will treat me differently if they know I am trans.”

The physicians interviewed expressed a need for this type of documentation. One provider said, “Primary care should always know if sex assigned at birth does not match preferred gender. Not knowing that can be a problem.” Another provider states, “There should be a place on the patient banner to represent GI, but we need a way to clue-in the provider to say it may not match sex at birth.” Clinicians argue that this information must be captured to guide care provision, particularly with regard to preventive screenings.

In addition to the scope of information, patients and physicians had differing opinions on who should be tasked with collecting GI information. Most clinicians interviewed said that birth-assigned sex and gender are typically collected on new patient intake forms and who collects this information is dependent on clinic workflow. For example, patient intake forms can be collected by registration staff or medical assistants. One provider said, “Pharmacy interns do all of the medical intakes on patients. Sex assigned at birth and GI should be collected by the intake interns.”

However, patients expressed uneasiness about who collects information regarding their GI. One patient said, “The physician would be the only person I would feel comfortable disclosing my GI to. It depends on the person, but I don't want to necessarily be outed as trans, especially by answering ‘not comfortable to disclose.’” A patient stated, “I would be willing to tell any person who *needed* to know, though I would feel most safe talking to somebody who received medical or social work training. I know that gender studies isn't always included in curriculum.” One patient summed up the safety concern by saying, “If you can't even go to the bathroom without fearing for your safety, then why would you feel safe disclosing your GI in a rural medical setting?”

## Discussion

Both patient and provider interviews offer us a glimpse into the structural difficulties of creating an EHR system that serves the needs of clinicians while providing safe and culturally competent care to the TGNC patients. Both groups would like the EHR to collect pronouns and names (even if different from legal name) and both agreed that these systems need to offer a broader range of birth-assigned sex and gender options. Providers and patients diverged over the need to capture birth-assigned sex and GI within every healthcare setting and who should be recording that information.

More research is needed to look at the reasons why patients and clinicians disagreed over the need to capture birth-assigned sex. Other TGNC research studies have shown similar results, but none have yet looked at why TGNC patients are hesitant to divulge that information.^5,32^ One patient commented in a 2014 study, “Though I understand the importance of knowing birth sex when dealing with trans medical issues, it's still a very sensitive question that most [transgender people] would probably not want to answer.”^32^ Follow-up interview questions or surveys could not only ask why patients are hesitant to answer these questions but also explore alternative ways to ask for this information.

Currently, neither MU nor UDSs have called for the collection of a patient's current name and pronouns, which both clinicians and patients agreed is important. Experts have noted that when the patient's name and pronouns are used incorrectly, it can be stigmatizing, create an environment that is not affirming, and cause the patient to avoid healthcare in the future.^32^ A lack of gender affirmation has been shown to adversely impact healthcare utilization behaviors, including delaying preventive healthcare screenings or avoiding needed clinical care when there is an acute need.^33,34^ Collecting GI sensitively and using it appropriately creates an environment of trust where patients feel safe disclosing and providers can use this information to guide clinical care. As there are no universal EHR products used in all health centers, it is important to not only research what patients and clinicians would like but to also help develop best practice guides for the collection of this information. Researchers should also work closely with EHR vendors to develop and improve their product offerings so that prompts and data collection fields are inclusive to TGNC individuals.

Limitations of this study include the small number of patients and providers interviewed. TGNC qualitative research can be hindered by small sample sizes as TGNC patients are often distrustful of healthcare systems and the number of providers that provide care to TGNC patients is limited.^35^ Thus, it was necessary for us to use convenience and targeted sampling that limits generalizability and may introduce selection bias.^35^ However, considering the economic realities of being TGNC in the United States, this study is an important contribution to the growing body of literature about how patients access care through the safety net.

## Conclusion

This qualitative work examines the complexity of GI collection in CHCs. While the collection of GI by MU and UDS is an important step for research designed to quantify health inequities, the data collection may not adequately guide care provision. The World Professional Association of Transgender Providers has established best practices that call for the collection of both birth-assigned sex and current GI and a means to maintain an inventory of a patient's medical transition history and current anatomy to guide care.^36^ Future research should focus on designing EHR tools across EHR vendors that not only collect GI data in the demographic fields but also guide clinical care. The TGNC patient's voice should help shape documentation processes and workflows to improve TGNC patient satisfaction and build trust with healthcare.

## Abbreviations Used

CHCs: community health centers
EHR: electronic health record
FQHCs: federally qualified health centers
GI: gender identity
IOM: Institute of Medicine
MU: meaningful use
ONC: Office of the National Coordinator for Health Information Technology
PHI: personal health information
TGNC: transgender and nonconforming
UDS: Uniform Data System
